# *Lithobius* (*Ezembius*) *zebrinus* sp. nov. (Lithobiomorpha, Lithobiidae), a new species from south-western China

**DOI:** 10.3897/BDJ.14.e194005

**Published:** 2026-07-02

**Authors:** Sujian Pei, Huiqin Ma, Bing Li, Zhida Chen, Kexin Zhang, Jiaqi Wang, Zhengyuan Zhang

**Affiliations:** 1 School of Life Sciences, Hengshui University, Hengshui, China School of Life Sciences, Hengshui University Hengshui China; 2 Hebei Key Laboratory of Wetland Ecology and Conservation of Hengshui University, Hengshui, China, Hengshui, China Hebei Key Laboratory of Wetland Ecology and Conservation of Hengshui University, Hengshui, China Hengshui China

**Keywords:** Chilopoda, new species, taxonomy, Yunnan

## Abstract

**Background:**

*Ezembius* was originally proposed as a subgenus of *Lithobius* Leach, 1814 in the family Lithobiidae in 1919 by Chamberlin; it accommodates a group of approximately 79 species/subspecies mostly known from Asia, with little extension into north-western North America.

**New information:**

A new species Lithobius (Ezembius) zebrinus sp. nov. is described, based on both sexes from Daweishan National Nature Reserve, Honghe Hani and Yi Autonomous Prefecture, Yunnan, south-western China. A detailed morphological description and illustrations of the new species are provided.

## Introduction

Known species of *Ezembius* colonise a wide range of habitats, from the Arctic and sub-Arctic regions to tropical and sub-tropical forests, from steppe and overgrazed stony areas of central Asia to Himalayan montane forests, from the seashore up to 5500 m (Himalayas) (Bonato et al. 2016, Qiao et al. 2018). Although the subgenus was formally proposed as new and described in 1923 (Chamberlin 1923), according to Jeekel (Jeekel 2005), its name was validated in 1919 (Chamberlin 1919). *Ezembius* is characterised by antennae with ca. 20 articles; ocelli 1+4–1+20; forcipular coxosternal teeth usually 2+2; porodonts generally setiform, sometimes stout. Tergites are generally without posterior triangular projections. Female gonopods are with uni-, bi- or tridentate claws and 2+2–3+3 (rarely 4+4) spurs (Zapparoli and Edgecombe 2011).

The myriapod fauna of China is still poorly known and this is especially the case with centipedes of the order Lithobiomorpha. In total, 114 species/subspecies of lithobiomorphs to date known from China. Altogether, 33 species of *Ezembius* have been recorded from China (Ma et al. 2014, Pei et al. 2014, Ma et al. 2015, Qiao et al. 2018, Qiao et al. 2019a, Qiao et al. 2019b, Chao et al. 2020a, Chao et al. 2020b, Zhang et al. 2026). South-western China represents one of the world’s biodiversity hotspots (Myers et al. 2000). However, taxonomy of Lithobiomorpha in this region are notably scarce, with only a single species hitherto documented in Yunnan Province (Chao et al. 2024). The present study represents a part of our investigation into Ezembius diversity in China; herein, a new species recently discovered in Daweishan National Nature Reserve, Honghe Hani and Yi Autonomous Prefecture, Yunnan, south-western China, is described and illustrated. Tables of the main morphological characters of Chinese Ezembius species are also presented.

## Materials and methods

All specimens was collected from leaf litter or under stones using entomological tweezers and preserved in 75% or 100% ethanol, respectively, for the purpose of meeting morphological and molecular biological research requirements. All specimens were examined using a ZEISS SteREO Discovery V20 microscope, equipped with an Abbe drawing tube and an ocular micrometer and Axiocam 512 colour. Plates and photographs were edited and retouched using Adobe Photoshop CS2. The external anatomical terminology follows the proposed terminology used in Bonato et al. (Bonato et al. 2010). The colour description was based on specimens preserved in 75% ethanol. Body length measured from the anterior margin of the cephalic plate to the posterior end of the postpedal tergite. Specimens have been deposited in Institute of Myriapodology, School of Life Sciences, Hengshui University, Hengshui, P. R. China (HUSLSIM).

Abbreviations are as follows: a: anterior; C: coxa; F: femur; m: median; p: posterior; P: prefemur; S, SS: sternite, sternites; T, TT: tergite, tergites; Tr: trochanter; Ti: tibia.

## Taxon treatments

### Lithobius (Ezembius) zebrinus

Pei, Ma & Li sp. nov.

29B88CA5-EEF1-5A24-8A31-5AF42EEB0B77

D15B47A3-1EDA-4723-9FE3-7C293F46EEEE

#### Materials

- **Type status:** Holotype. **Occurrence:** recordedBy: Ma Huiqin; individualCount: 1; sex: male; lifeStage: adult; occurrenceID: E2DE4597-B582-5E71-A648-218F523BE6DA; **Taxon:** scientificName: Lithobius (Ezembius) zebrinus sp. nov.; class: Chilopoda; order: Lithobiomorpha; family: Lithobiidae; genus: Lithobius; subgenus: Ezembius; **Location:** country: China; stateProvince: Yunnan; locality: Daweishan National Nature Reserve; verbatimElevation: 1080 m; verbatimCoordinates: N22°45′59.00″ E103°04′57.00″; **Identification:** identifiedBy: Ma Huiqin; dateIdentified: 2025; **Event:** eventDate: 24/01/2019; **Record Level:** collectionCode: Myriapoda; basisOfRecord: Preserved Specimen
- **Type status:** Paratype. **Occurrence:** recordedBy: Ma Huiqin; individualCount: 3; sex: male; lifeStage: adult; occurrenceID: C99ABEE9-5952-563C-87D1-1A7804D79F4B; **Taxon:** scientificName: Lithobius (Ezembius) zebrinus sp. nov.; class: Chilopoda; order: Lithobiomorpha; family: Lithobiidae; genus: Lithobius; subgenus: Ezembius; **Location:** country: China; stateProvince: Yunnan; locality: Daweishan National Nature Reserve; verbatimElevation: 1080 m; verbatimCoordinates: N22°45′59.00″ E103°04′57.00″; **Identification:** identifiedBy: Ma Huiqin; dateIdentified: 2025; **Event:** eventDate: 24/01/2019; **Record Level:** collectionCode: Myriapoda; basisOfRecord: Preserved Specimen
- **Type status:** Paratype. **Occurrence:** recordedBy: Ma Huiqin; individualCount: 7; sex: female; lifeStage: adult; occurrenceID: 75D4E9F1-6B88-5786-A3BA-EB71ADDE2A5B; **Taxon:** class: Chilopoda; order: Lithobiomorpha; family: Lithobiidae; genus: Lithobius; subgenus: Ezembius; **Location:** country: China; stateProvince: Yunnan; locality: Daweishan National Nature Reserve; verbatimElevation: 1080 m; verbatimCoordinates: N22°45′59.00″ E103°04′57.00″; **Identification:** identifiedBy: Ma Huiqin; dateIdentified: 2025; **Event:** eventDate: 24/01/2019; **Record Level:** collectionCode: Myriapoda; basisOfRecord: Preserved Specimen

#### Description

**Figure 1.**
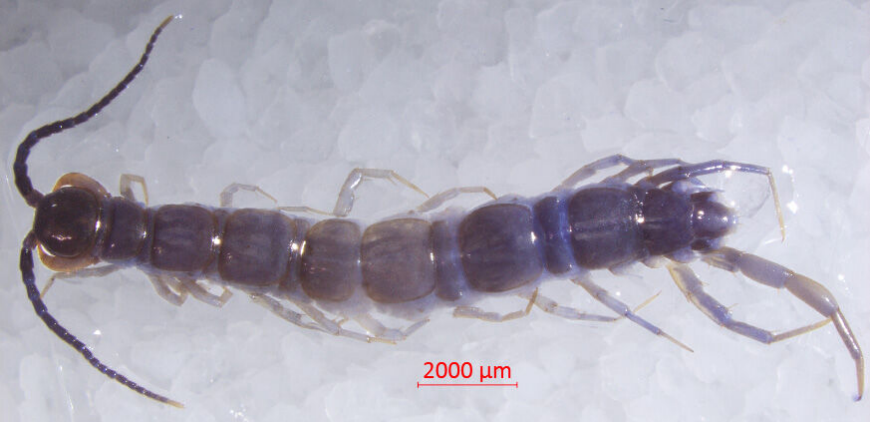
Lithobius (Ezembius) zebrinus sp. nov., male holotype, habitus, dorsal view.

**Body**: Measurements. Male holotype body 12.39 mm long, cephalic plate 1.56 mm long, 1.50 mm wide. Body length 12.39–15.06 mm. cephalic plate length 1.33–1.61 mm, width 1.10–1.56 mm (Fig. 1).

**Colour**: The antennae mostly brown, darkest at the basal segments, lightest at the terminal segments, appearing pale yellow, with pale grey at the junctions between segments. Tergites dark brown, with the middle ones (TT 7, 8) being the lightest, approaching pale yellow. Pleural region light grey to dark grey. The sternites light yellowish–brown. The head shield, coxosternite and forcipular tarsungulum dark yellowish-brown, while the rest of the forcipular tarsungulum fulvous to testaceous, coxosternite, S 15 and ventral plate of the pregenital segment yellowish–brown. The coxae and tarsi of all legs yellowish–brown, occasionally with pale grey at the base, with the ta2 darker and the middle segments are pale grey.

**Antennae**: Mostly 18–22 articles (few 24), holotype male 20+20 articles. The first basal article significantly longer than wide, the length of the subsequent articles gradually decreasing, with the final article significantly longer than wide, 3.1–3.4 times longer than wide (Fig. 1); abundant setae on the antennal surface, fewer on the basal articles, gradual increasing in density to approximately the sixth or seventh article, then more or less constant.

**Figure 2.**
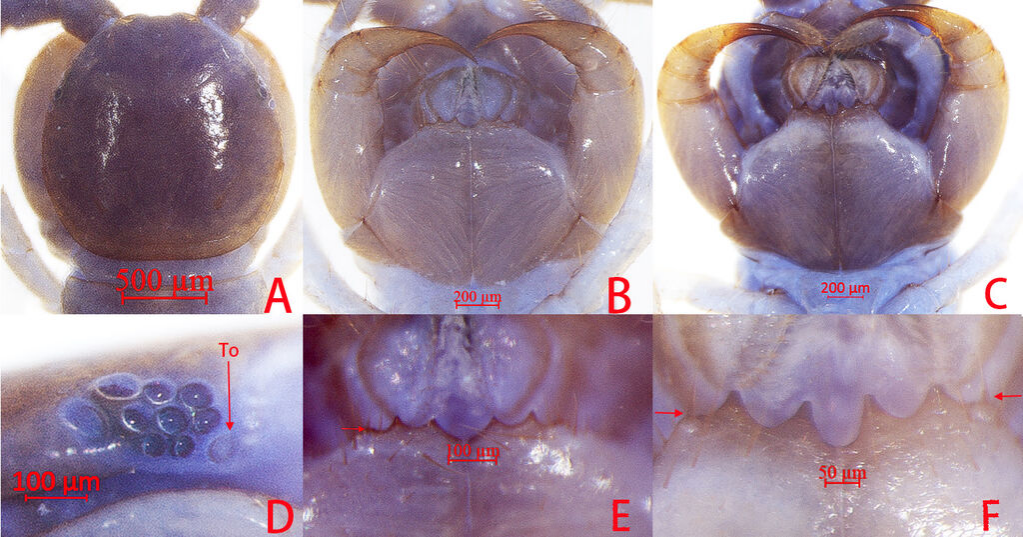
Lithobius (Ezembius) zebrinus sp. nov., **A** cephalic plate, dorsal view; **B** (female)–**C** (holotype) forcipular coxosternite, ventral view; **D** ocelli and Tömösváry’s organ (To); **E** (female)–**F** (holotype) forcipular coxosternite, ventral view.

**Cephalic plate**: Smooth, convex, slightly longer than broad, tiny setae scattered sparsely over the whole surface, the margins of the cephalic plate with very sparsely distributed, nearly uniformly long setae and the whole surface of cephalic plate is covered with a fine hexagonal mesh. Anterior margin of the cephalic plate slightly concave central, with a distinct transverse groove, the lateral margin of the cephalic capsule only beginning with a ridge from the side to the mid-point between the ocelli and the posterior margin, with a slightly convex posterior margin and a complete ridge (Fig. 2 A).

**Ocelli**: 7–9 ocelli, usually 8, arranged in three irregular rows, oval to rounded ocelli on each side, the posterior ocellus larger, the ventral ocelli are smaller than the dorsal ocelli in size. Ocelli mostly translucent, with black pigmentation at the base and hemispherical in shape (Fig. 2D).

**Tömösváry’s organ**: Close to the ocelli, situated at the anterolateral margin of the cephalic plate, on the ventral side of the anterior part of the eye area, slightly yellow in the perimeter. The Tömösváry’s organ small, nearly round, the surrounding sclerotised area narrow and slightly larger than the adjoining ocelli (Fig. 2D).

**Coxosternite**: Subsemicircular in female (Fig. 2B), the lateral and anterior margins grade imperceptibly into one another, median diastema moderately shallow, V-shaped; subtrapezoidal in male (Fig. 2C), lateral margins moderately shorter than the medial margins. Anterior margin narrow, median diastema moderately deep, U-shaped. Whole surface of coxosternite covered with fine hexagonal mesh. Anterior margin bearing 2+2 blunt triangular teeth, porodonts slightly thicker, almost transparent, lying posterolateral to and separated from the lateral-most tooth, with slightly basal bulge (Fig. 2E and F). The surface of the coxosternite bears prominent striae arranged in bilateral symmetry. Near the anterior margin of the coxosternite, two to three striae run substantially straight. In the transitional zone extending from the mid-region to the vicinity of the posterior margin, the angle between the striae and the mid-line progressively decreases, while the overall pattern exhibits a radial convergence curving inwards from the lateral regions toward the mid-line (Fig. 2B and C). Scattered long setae on the ventral side of coxosternite, longer setae near the dental margin.

**Tergites**: Smooth, slightly convex; short to long tiny setae scattered sparsely over the entire surface, near the margin with more long setae; T1 narrower than the cephalic plate and T3, cephalic plate wider than T3. T1 narrower postero-laterally than antero-laterally, generally inverted trapezoidal. The lateral marginal ridges of all tergites continuous, posterior marginal ridge of TT1 and 3 continuous, posterior marginal ridge of TT5, 8, 10, 12 and 14 discontinuous. The posterior margin of TT1 and 3 straight, posterior margin of TT5, 7, 8 slightly concave, posterior margin of TT7, 8, 10, 12 and 14 concave. The posterior angles of all tergites without triangular projections.

**Sternites**: Posterior margin of sternites narrower than anterior, generally inverted trapezoidal, smooth; long setae on the surface and lateral margin, very few short setae scattered sparsely amongst them, with 1–3 longer setae at the anterior corners and 2–13 longer setae located in the centre of the anterior part, arranged approximately in two rows.

**Legs**: Relatively robust, tarsi well-defined on legs 1–15. All legs with moderately long curved claws. Legs 1–15 each carry an anterior accessory spur and a posterior accessory spur: the anterior spur moderately longer and slender, forming a moderately small angle with the claw; the posterior spur slightly longer and more robust, forming a comparatively large angle with the claw. Short to long setae very sparsely scattered over the surface of the coxa, trochanter, prefemur, femur and tibia of all legs. The setae are shorter and sparser from the coxa to the profemur, becoming progressively longer and denser from the femur distally to the tarsus, with the longest setae occurring on the tarsus. Furthermore, the setae on the anterior and posterior surfaces are shorter than those on the dorsal and ventral surfaces. Legs 14 and 15 obvious longer and thicker than the anterior legs, without secondary sexual characters in both female and male; tarsus 2, 8.48–9.05 times longer than wide, tarsus 2, 63.1%–67.3% length of tarsus 1 on leg 15 in female; tarsus 2 5.15–5.72 times longer than wide, tarsus 2, 63.1%–67.3% length of tarsus 1 on leg 15 in male. Leg plectrotaxy given in Table 1 and Table 2.

**Coxal pores**: 3–5 in number, subcircular, markedly unequal in size. Female pattern predominantly 45,5,5; male pattern 4,4,4,3. The coxal pore field hardly sunken, forming a shallow groove with elevated margins that bear sparsely distributed setae of varying lengths.

**Figure 3.**
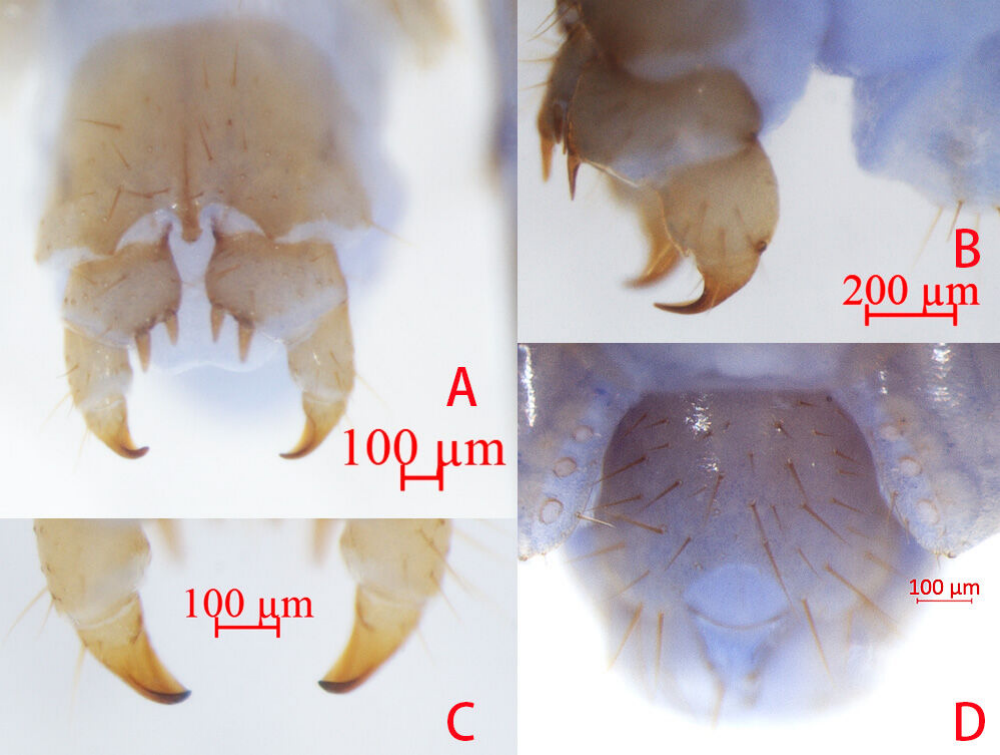
Lithobius (Ezembius) zebrinus sp. nov. **A** postpedal segments and female gonopods, ventral view; **B** female gonopods, lateral view; **C** apical claw of female gonopods, magnified ventral view; **D** (holotype) posterior segments and male gonopods, ventral view.

**Female**: S15 anterior margin broader than posterior, generally nearly semi-circular, posterior angles rounded, postero-medially straight. Moderately long to short setae relatively sparsely scattered on S15 surface. Broader than length, surface of the lateral sternal margin of pregenital segment well chitinised, posterior margin of pregenital sternite deeply concave between condyles of gonopods, except for a small, median lingulate-shaped bulge. Setae moderately densely scattered over ventral surface of the pregenital segment, slightly more setae on posterior part, especially along the posterior edge. Gonopods: first article fairly broad, bearing 25–35 moderately long setae, arranged in approximately four irregular longitudinal rows, with 2+2 small blunt coniform gonopodal spurs; second article with 5–9 long setae in the ventral, arranged in approximately three irregular rows and 1–3 setae dorsally in a longitudinal row. Third article with 2–4 longer setae on the ventral surface, arranged in two rows, with a simple apical claw (Fig. 3A, B and C).

**Male**: S15 posterior margin narrower than anterior, postero-medially slightly concave, generally an inverted trapezoid, sparsely covered with short to long setae, the setae on the edges being longer; the pregenital sternite segment evidently smaller than the female, usually well sclerotised, ventral side obviously convex; posterior margin shallow concave between the gonopods, without medial bulge. Short to long setae equally scattered on the ventral surface of the pregenital segment. Gonopods short and small, papilliform, with four long setae, apically, slightly sclerotised (Fig. 3D).

#### Diagnosis

In accordance with the grouping of species proposed in the subgenus Ezembius Chamberlin, 1919, the new species differs from other consubgeners in combination of characters, by having the surface of the coxosternite bearing prominent striae arranged in bilateral symmetry, the coxosternite of males and females differing markedly in morphology, the antennae 18–22 articles, ocelli 7–9 on each side, arranged in three rows, with the last one ocellus being the largest, Tömösváry’s organ slightly larger than the adjacent ocelli; commonly 2+2 coxosternal teeth; coxal pore formula 3–5. Female gonopods with 2+2 spurs, apical claw of the third article simple, male gonopods short and small, papilliform, with four long setae.

#### Etymology

The specific epithet, meaning the distinct transverse striae on the ventral side of the coxosternite.

#### Taxon discussion

Morphologically, the new species is close to L. (E.) datongensis Qiao, Qin, Ma, Zhang, Su & Lin, 2018 from Datong County, Qinghai Province, in having Tömösváry’s organ larger than the adjoining ocelli, commonly 2+2 coxosternal teeth, female gonopods with 2+2 spurs, apical claw of the third article simple. It can be readily distinguished by: ocelli 7–9 on each side versus 10 on each side in L. (E.) datongensis; leg 15 bearing both anterior and posterior accessory spurs versus only a posterior spur in L. (E.) datongensis; the apical claw of the female gonopods simple versus bears a distinct basal triangular protuberance in L. (E.) datongensis; coxae of legs 13–15 without anterior spines versus with anterior spines in L. (E.) datongensis.

The new species and L. (E.) hualongensis Qiao, Qin, Ma, Lin & Zhang, 2019 from Hualong Hui Autonomous County, Qinghai Province, share ocelli arranged in irregular three rows, Tömösváry’s organ larger than the adjoining ocelli, commonly 2+2 coxosternal teeth and apical claw of female gonopods simple. Diagnostic differences are: the posterior ocellus larger versus the posterior two ocelli larger in L. (E.) hualongensis; legs 14 and 15 each carry an anterior accessory spur and a posterior accessory spur versus leg 14 carrying a posterior accessory spur and leg 15 without accessory spur in L. (E.) hualongensis; female gonopods with 2+2 spurs versus 3+3 spurs in L. (E.) hualongensis; legs 13–15 without anterior spines on the dorsal side of the coxae with anterior spines in L. (E.) hualongensis.

The new species closely resembles L. (E.) hirsutipes Eason, 1989 from Xizang Autonomous Region, in possessing antennae 20 + 20 articles, ocelli arranged in irregular three rows, 2+2 coxosternal teeth, female gonopods bearing 2+2 spurs. It is readily distinguished by: ocelli 7–9 on each side versus ocelli 10–11 in L. (E.) hirsutipes; the coxosternite of males and females differ markedly in morphology versus substantially similar between sexes in L. (E.) hirsutipes, the second article of the female gonopods lacking dorsal robust spines versus possessing five robust spines in L. (E.) hirsutipes; and the apical claw of the third article of the female gonopods simple and with a very faint ventral tubercle in L. (E.) hirsutipes; and the absence of anterior dorsal spines on the coxae of legs 13–15 versus present in L. (E.) hirsutipes.

#### Notes

To facilitate the identification of Chinese subgenus Ezembius Chamberlin, 1919 species, the distribution and main morphological characters of the known species of the subgenus in the area is presented (Tables 3, 4, 5, 6, 7). These characters are specific only to adults of the taxa occurring in China.

## Supplementary Material

XML Treatment for Lithobius (Ezembius) zebrinus

## Figures and Tables

**Table 1.** Leg plectrotaxy of Lithobius (Ezembius) zebrinus sp. nov., males.

Legs	Ventral					Dorsal				
	C	Tr	P	F	Ti	C	Tr	P	F	Ti
1			p		m			p	ap	a
2			p	amp	m			p	ap	a
3			p	amp	m			p	ap	a
4			p	amp	m			p	ap	ap
5-6			mp	amp	am			p	ap	ap
7-10			mp	amp	am			ap	ap	ap
11			mp	amp	am			amp	ap	ap
12			amp	amp	am			amp	ap	ap
13		m	amp	amp	am			amp	ap	ap
14		m	amp	am	am			amp	ap	ap
15		m	amp	am	a			amp	ap	

**Table 2.** Leg plectrotaxy of Lithobius (Ezembius) zebrinus sp. nov., females.

Legs	Ventral					Dorsal				
	C	Tr	P	F	Ti	C	Tr	P	F	Ti
1					m				a	a
2			p	(a)m	m			(a)p	a	a
3			p	(a)m	(a)m			p	a(p)	a(p)
4-5			p	(a)m	m			p	ap	a
6-7			mp	(a)m	(a)m			ap	ap	a(p)
8-10			mp	am	m			ap	ap	ap
11			mp	amp	am			amp	ap	ap
12			mp	amp	am			ap	ap	ap
13		m	mp	(a)mp	am			amp	p	
14		m	amp	am	am			amp	p	
15		m	amp	am				amp	a	

N.B. Letters in brackets indicate variable spines.

**Table 3.** The main morphological characters of the known Chinese species of subgenus Ezembius Chamberlin, 1919.

**characters**	***anabilineatus***	***anasulcifemoralis***	***bidens***	***bilineatus***	***brachyspinipes***	***chekianus***	***daliensis***
Authorities	Ma et al., 2015 (Ma et al. 2015)	Ma et al., 2013 (Ma et al. 2013)	Takakuwa, 1939 (Takakuwa 1939)	Pei et al., 2014 (Ma et al. 2014)	Pei et al., 2020 (Pei et al. 2020)	Chamberlin and Wang, 1952 (Chamberlin and Wang 1952)	Chao et al., 2024 (Chao et al. 2024)
Distribution	China S (Guangxi)	China S (Guangxi)	China S (Taiwan)	China S (Guangxi)	China N(Henan)	China E (Zhejiang)	China SW (yunnan)
Body length (mm)	11.9–12.1	10.1–12.3	15	9.0–9.1	9.6–13.3	16	18.0–22.0
Number of antennal articles	23+23 articles in female, unkown in male	19–24, commonly 20+20	20–21	two specimens with 20+21, one specimen with 20+23	21–24	20＋20	20 or 21
Number, arrangement and shape of the ocelli	5 – 6, in 3 rows	6, in 3 rows	7	5–6, in 2 rows	8–10, in 2 or 3 rows	5, in 2 rows	9 or 10, in 3 irregular rows
Posterior ocellus	round, large	oval to round, large	comparatively large	oval to rounded	oval to rounded, comparatively large	oval to round, comparatively large	the most posterior ocellus largest
Tömösváry's organ	round, smaller than adjoining ocelli	moderately large, rounded, smaller than adjoining ocelli	at most same size as one ocellus	slightly larger than adjoining ocelli	smaller than the adjacent ocelli	not reported	smaller than the adjoining ocelli
Number and arrangement of coxosternal teeth	2+2, subtriangular	2+2, subtriangular	2+2	2+2, slightly triangular	2+2, subtriangular slightly acute teeth	2+2	2+2, subtriangular teeth
Number of coxal pores	3–5, male 4454, 3554; female 4443, 4453	3–6, usually 4663, 5654, 5553,5563 and 5565	5(6)555	usually females 4554, 5565; males 4553, 4454	3–6, usually 4453, 5553, 4554, 5554	6655 or 7665	5555 or 6555
Male 14 leg	obvious, thicker and stronger than other legs	markedly thicker and stronger than legs 1–13	not reported	distinctly thick and strong	significantly thicker	not reported	not reported
Male 15 leg	obvious thicker and stronger than other legs	markedly thicker and stronger than legs 1–13	not reported	distinctly thick and strong	significantly thicker	not reported	thicker and stronger
DaC spine	on legs 14–15	on legs 14–15	absent	on legs 14–15	on legs 13–15	on legs 14–15	on legs 14–15
14 leg accessory spur	the anterior reduced in size, only half length of the posterior	absent	with a pair of accessory claws	anterior accessory spur absent	both present	present	present
15 leg accessory spur	absent	absent	with visible accessory spur	anterior accessory spur absent	absent	present	present
Number and shape of spurs on female gonopods	2+2 moderately small, blunt, coniform, inner spur slightly smaller than the outer	2+2 moderately blunt, with conical spurs, inner spur slightly smaller	3+3 or 4+4, sharp	2+2, moderately small, blunt, coniform, inner spur slightly smaller than outer one	2+2, moderately small, blunt, coniform, inner spur slightly smaller than outer one	not reported	2+2, 3+2 or 3+3; sharp coniform spurs, inner spur smaller
Apical claw of female gonopods (and lateral denticles)	simple, small subtriangular teeth in the inner	apical claw dimidiate	not reported	apical claw bipartite	two robust spines, apical claw bipartite	not reported	undivided, a small sharp lateral denticle on base of terminal claw
Male gonopods	short and small bulge, with one long setae	with a small bulge, without setae	hemispherical, with two long setae	short and small bulge, having a long seta	hemispherical bulge	not reported	short and small, with 2–4 long setae, slightly chitinized apically

**Table 4.** The main morphological characters of the known Chinese species of subgenus Ezembius Chamberlin, 1919.

**characters**	***datongensis***	***dulanensis***	***gantoensis***	***helanensis***	***hirsutipes***	***hualongensis***	***insolitus***
Authorities	Qiao et al., 2018 (Qiao et al. 2018)	Qiao et al., 2019 (Qiao et al. 2019b)	Takakuwa and Takashima, 1949 (Takakuwa and Takashima 1949)	Lu et al., 2022 (Lu et al. 2022)	Eason, 1989 (Eason 1989)	Qiao et al., 2019 (Qiao et al. 2019a)	Eason, 1993 (Eason 1993)
Distribution	China NW (Qinghai)	China NW (Qinghai)	China N (Shanxi)	China NW (Ningxia)	China NW (Xizang)	China NW (Qinghai)	China S (Hong Kong)
Body length (mm)	12.3–14.2	20.5	9.0	17.6–29.8	12.7–15.7	12.3–16.2	10.0－11.5
Number of antennal articles	20+20	20–21	20–23	19-28, commonly 21+21	20-20	20+20	18+18－19+19
Number, arrangement and shape of the ocelli	10, in 3 rows	11–12, in 3 rows	6, in 2 rows	9-16, commonly 12–14; in 3, 4 or 5 rows	10-11, in 3 rows	8–11, in 3 irregular rows	6–8, in 2 rows
Posterior ocellus	comparativelylarge	ovate to circular, comparatively large	not reported	the most posterior ocellus largest	comparatively large	two ocelli comparatively large	comparatively large
Tömösváry's organ	slightly largerthan nearest ocellus	slightly smaller than the adjoining ocelli	subequal in size to adjoining medium large ocelli	same size as adjacent ocelli	same size as the largest ocellus	larger than the adjoining ocelli	slightly smaller than adjoining ocelli
Number and arrangement of coxosternal teeth	2+2, slightly acute	2+2 moderately robust teeth	2+2, approximately sharp, small	commonly 2+2, few 2+3, very few 3+3, 3+4, 4+5; subtriangular slightly acute teeth	2+2, small and subtriangular slightly acute teeth	2+2, small blunt teeth	2+2
Number of coxal pores	female 4655, 5575 and 5544. Coxal pores 4654 and 4554 in male	5667 or 5666	3333	6-10, commonly 7 or 8	4-6, female 5555, 4555; male 5555, 4555, 4554, 4544, 4444	round or slightly oval, 6666 and 6777 in female and 4654 and 4655 in male	3–6, male 3443; female 4454, 4555, 5555, 5565
Male 14 leg	significantly thicker	longer and thicker than legs 1–13	not reported	thick and strong	thicker and stronger	thicker and stronger	distinctly thick and strong
Male 15 leg	significantly thicker	longer and thicker than legs 1–13	not reported	thick and strong	thicker and stronger	thicker and stronger	distinctly thick and strong, with dark zones on dorsal of tibia
DaC spine	on legs 12–15	on legs 11–15	absent	on legs 12–15	on legs 11–15	on legs 12–15	absent
14 leg accessory spur	present	anterior accessory spines absent	present	present	present	with only posterior spines	not reported
15 leg accessory spur	anterior accessory spur absent	absent	present	only posterior accessory spur	absent	absent	absent
Number and shape of spurs on female gonopods	2+2 moderately large, coniform spurs	2+2, moderately small coniform spurs	1+1, apically acute	2-3, commonly 3+3 few 2+3; small, coniform spurs, spurs, inner spur smaller	2+2, coniform spurs, the inner ones smaller and acuter, the outer ones larger and blunter	3+3, moderately long and slender spurs	3+3, coniform spurs
Apical claw of female gonopods (and lateral denticles)	undivided, bearing a small triangular protuberance on ventral side	simple, curved	simple	simple and broad	slender and sharp, having a relatively small triangular protuberance on the dorsal side, and a weak protuberance on the ventral side	simple and sharp, having a very small triangular protuberance on ventral side	simple
Male gonopods	hemispherical bulge, with three setae	small, one-segmented, with two long setae	not reported	short, a small hemispherical bulge, with 0-2 long setae	short, appearing as a small hemispherical bulge, with 2-4 long setae	short, appearing as a small hemispherical bulge, with one long setae	not reported

**Table 5.** The main morphological characters of the known Chinese species of subgenus Ezembius Chamberlin, 1919.

**characters**	***irregularis***	***keelungensis***	***kiayiensis***	***laevidentata***	***lineatus***	***longibasitarsus***	***mandschreiensis***
Authorities	Takakuwa and Takashima, 1949 (Takakuwa and Takashima 1949)	Chao et al., 2020 (Chao et al. 2020a)	Wang, 1959 (Wang 1959)	Pei et al., 2015 (Pei et al. 2015)	Takakuwa, 1939 (Takakuwa 1939)	Qiao et al., 2018 (Qiao et al. 2018)	Takakuwa, 1939 (Takakuwa 1939)
Distribution	China N (Shanxi)	China S (Taiwan)	China S (Taiwan)	China NW (Xinjiang Uygur)	China S (Taiwan)	China NW (Qinghai)	China (Taiwan, Sichuan, Hebei, Jiangsu, Heilongjiang, Jilin, Liaoning)
Body length (mm)	12.0	11.0–13.5	9.0–12.0	9.6–13.3	18.0	17.0–18.0	22.0–23.0
Number of antennal articles	20+20	20	26-31	19–22 commonly 20+20, only one specimen 25+20	19+19–21+21	20+20	20–28
Number, arrangement and shape of the ocelli	7	7–9, in 3 irregular rows	6–8	8–10, in 3 rows	8–10, in 3 rows	10–14, in 3 irregular rows	9–12, in 3 rows
Posterior ocellus	comparatively large	posterior ocellus largest	not reported	posterior two ocelli bigger than seriate ocelli	not reported	posterior ocellus largest	same size
Tömösváry's organ	same size as the largest ocellus	larger than adjoining ocelli	not reported	subequal in size to adjoining ocelli	same size as adjoining ocelli	smaller than adjoining ocelli	larger than adjoining ocelli
Number and arrangement of coxosternal teeth	2+2, small	2+2, large triangular, the inner are larger than the outer	3+3	2+2, most are relatively sharp, while a few are rather blunt and rounded	2+2, comparatively large	2+2 or 2+3 blunt nipple-like teeth	2+2, small and sharp
Number of coxal pores	3–10, 3–6 in 12 leg, 4–6 in 13 leg, 7–10 in 14 and 15 leg	4–6	4444	2–5, female commonly 4555, 4554, sometime 3454, 3455, 3343. male 2332, 2333, 3444, 3333	6–7, usually 6(7)666	4–6; 5544, 5554 or 6555	776(7)5(6)
Male 14 leg	not reported	distinctly thick	not reported	remarkably thicker and stronger	not reported	moderately thicker and longer	not reported
Male 15 leg	not reported	distinctly thick	not reported	markedly thicker and stronger	not reported	moderately thicker and longer	not reported
DaC spine	on legs 13–15	absent	absent	on legs 12–15	on legs 14–15	on legs 13–15, 12 sometimes present	on legs 12–15
leg 14 accessory spur	not reported	very short anterior accessory spurs	not reported	present	present	present	not reported
leg 15 accessory spur	not reported	anterior absent	not reported	absent	present	absent	not reported
Number and shape of spurs on female gonopods	2+2 or 2+3, the basal spurs are large, short and sharp	2+2, sharp coniform spurs, the inner are larger than the outer	2+2	3+4, or 4+4 small, blunt, coniform spurs, commonly with 3+3, inner spur smaller than outer one	3+3 moderately sharp, slender conical spurs	2+2 moderately long, bullet-shaped spurs inner spur slightly smaller and more anterior than outer one	3+3, same size
Apical claw of female gonopods (and lateral denticles)	simple	undivided, a small sharp lateral denticle on the base of the terminal claw	tridentate	simple, a small approximately triangular process on the ventral side of the base	simple	simple, having small triangular protuberance on ventral side	simple
Male gonopods	not reported	short and small, as a semispherical bulge with 3 long setae, apically well chitinized	not reported	small bulge, with one to two long setae	hemispherical bulge	small, semicircular with 3-5 seta on its surface	without setae

**Table 6.** The main morphological characters of the known Chinese species of subgenus Ezembius Chamberlin, 1919.

**characters**	***maqinensis***	***multispinipes***	***parvicornis***	***polyommatus***	***potanini***	***rapax***	***rhysus***
Authorities	Qiao et al., 2019 (Qiao et al. 2019a)	Pei et al., 2016 (Pei et al. 2016)	Porat, 1893 (Porat 1893; Zapparoli 1991)	Qiao et al., 2019 (Qiao et al. 2019b)	Sseliwanoff, 1881 (Sseliwanoff 1881)	Meinert, 1872 (Meinert 1872)	Attems, 1934 (Attems 1934)
Distribution	China NW (Qinghai)	China NW (Xinjiang Uygur)	China S (Taiwan)	China NW (Xizang)	China NW (Xinjiang Uygur)	China S (Taiwan)	China S (Fujian and Taiwan)
Body length (mm)	13.1–14.6	famale 11.6–22.6, male 14.3–19.6	16.0	16.1–18.3	13.0–15.0	not reported	15.0
Number of antennal articles	20+20	commonly 20+20, (three specimens with 20+21, one specimen with 20+26 of 133 specimens)	20+20, 21+21	20+20	20 or 23	19–22	20+20 in female, 21+21 in male
Number, arrangement and shape of the ocelli	9–12, in 3 irregular rows	8, in 3 rows	3–4, in 1 or 2 rows	14, in 3 or 4 rows	8–10	7, in 2 rows	8, in 4 rows
Posterior ocellus	the most posterior ocellus largest	two ocelli large, oval to rounded	comparatively large	comparatively large	not reported	comparatively large	comparatively large
Tömösváry's organ	almost the same size as adjacent ocelli	slightly smaller than adjoining ocelli	not reported	moderately smaller than the adjoining ocelli	smaller than the adjacent ocelli	not reported	not reported
Number and arrangement of coxosternal teeth	2+2	2+2 slightly triangular	2+2	2+2 subtriangular slightly acute teeth	2+2, small and sharp	2+2	2+2
Number of coxal pores	6666	3–5, 4555, 5555, 4444, 4455 (females) and 4444, 3344 (males)	3334	4–7, 5676, 5666 (females) 5565, 4554 (males)	3–4	4554	6554
Male 14 leg	longer and thicker	thick and strong	not reported	significantly thicker and stronger	not reported	not reported	not reported
Male 15 leg	longer and thicker than legs 1–13	thick and strong	not reported	significantly thicker and stronger	not reported	femur and tibia thicker	prefemur and femur thicker
DaC spine	on legs 12–15, 11 sometimes present	on legs 11–15, 9–10 sometimes present	not reported	on legs 11–15	not reported	on leg 15 present	on leg 15 present
14 leg accessory spur	posterior accessory spurs present	present	not reported	with anterior and posterior accessory spurs	not reported	not reported	not reported
15 leg accessory spur	absent	absent	not reported	absent	not reported	not reported	absent
Number and shape of spurs on female gonopods	2+2 moderately small, coniform spurs, spurs, inner spur smaller	2+2, blunt, coniform spurs, with inner spur smaller than outer one	2+2	2+2 moderately long and slender, bullet-shape spurs	2 spurs, widely separated	2+2, thick spurs	2+2, slender
Apical claw of female gonopods (and lateral denticles)	unidentate, curved with a small triangular protuberance on ventral side	simple	simple	simple	simple, 1 stout spurs arranged in no regular pattern	dimidiate	simple
Male gonopods	small, undivided, oblique apically, with 2 setae	hemispherical bulge, having 2 long seta	not reported	short, a small hemispherical bulge with 2 long setae	small, with 1 long setae	not reported	not reported

**Table 7.** The main morphological characters of the known Chinese species of subgenus Ezembius Chamberlin, 1919.

**characters**	***Sui***	***sulcifemoralis***	***ternidentatus***	***varioporus***	***Zebrinus*** **sp.nov.**	***zhui***
Authorities	Qiao et al., 2019 (Qiao et al. 2019a)	Takakuwa and Takashima, 1949 (Takakuwa 1939)	Pei et al., 2019 (Pei et al. 2019)	Pei et al., 2020 (Pei et al. 2020)		Pei et al., 2011 (Pei et al. 2011)
Distribution	China NW (Qinghai)	China N (Shanxi)	China N (Hebei)	China N (Hebei)	China SW (yunnan)	China NW (Xinjiang Uygur)
Body length (mm)	12.2–18.9	12.0	7.1–8.5	12.4–13.6	12.4–15.1	8.1–15.0
Number of antennal articles	20+20	20+20	22-25, commonly 24, few 22+24 or 24+25	20–22, commonly 20+20, few 20+21 or 20+22	18–22, few 24	20－24, commonly 20
Number, arrangement and shape of the ocelli	9–10, in 3 irregular rows	6	5–6, in 2 irregular rows	9–10, in 3 rows	7–9, in 3 rows	10–13, in 3–4 rows
Posterior ocellus	two ocelli large	all ocelli same size	two ocelli comparatively large	two ocelli comparatively large	comparatively large	comparatively large
Tömösváry's organ	larger than the adjacent ocelli	same size as ocelli	same size as the largest two ocelli	largerthan nearest ocellus	slightly larger than the adjacent ocelli	slightly larger than adjoining ocelli
Number and arrangement of coxosternal teeth	3+3, subtriangular bluntly rounded teeth	2+2, small and sharp	commonly 3+3, but also 3+2 or 2+2	2+2, subtriangular slightly obtuse teeth	2+2, subtriangular slightly acute teeth	2+2, moderately small and pointed
Number of coxal pores	4–8, 5664, 5665, 7775, 8875 in female, and 6886, 7665 in male.	5555	33(4)4(5)3, commonly 3443	3-8, females 4554, 67(8)7(8)6, 5(6)765, 6(7)776, 66(7)65; males 66(7)7(6)5(4), 565(6)3	3-5, female 4555, male 4443	2–4, 3444, 3344, 3443, 3333 in female, and 3443, 2343, 2433, 2333 in male.
Male 14 leg	thicker and stronger	thick and strong	moderately thicker and longer	moderately thicker and stronger	moderately thicker and stronger	moderately thicker and stronger
Male 15 leg	thicker and stronger	thick and strong	moderately thicker and longer	moderately thicker and stronger	moderately thicker and stronger	moderately thicker and stronger
DaC spine	on legs 12–15	absent	on legs 10–15	absent	absent	on legs 13–15, 12 sometimes present
14 leg accessory spur	only posterior accessory spur	not reported	small posterior accessory spurs	only anterior spines	present	present
15 leg accessory spur	only posterior accessory spur	not reported	small posterior accessory spurs	only anterior accessory spur	present	absent
Number and shape of spurs on female gonopods	3+3, moderately long and slender, bullet-shape spurs	2+2, strong, long and sharp	2+2, moderately long and slender, coniform spurs	3+3, small coniform spurs, inner spur slightly smaller than outer one	2+2, bullet-shape spurs	2+2 moderately long, coniform spurs
Apical claw of female gonopods (and lateral denticles)	simple and sharp, having a very small triangular protuberance on ventral side	simple	simple	simple and broad	simple,with 2–4 setae	broad, and tridentate
Male gonopods	short, a small hemispherical bulge, with 2 long setae	with a few setae	short, a small hemispherical bulge, with 1–3 long setae	short, a small finger-like bulges, with 3–4 long setae	small bulge, with 4 long setae	small bulge, with 1–2 long setae
